# Surveying predictors of late-life longitudinal change in daily activity energy expenditure

**DOI:** 10.1371/journal.pone.0186289

**Published:** 2017-10-17

**Authors:** Vincenzo Valiani, Sandrine Sourdet, Dale A. Schoeller, Dawn C. Mackey, Douglas C. Bauer, Nancy W. Glynn, Yosuke Yamada, Tamara B. Harris, Todd M. Manini, for the Health, Aging and Body Composition Study

**Affiliations:** 1 Department of Aging and Geriatric Research, University of Florida, Gainesville, Forida, United States of America; 2 Dipartimento Interdisciplinare di Medicina, Clinica Medica Cesare Frugoni, University of Bari Aldo Moro, Bari, Italy; 3 Gérontopôle, Hôpital La Grave-Casselardit, Toulouse, France; 4 Department of Nutritional Sciences, University of Wisconsin-Madison, Madison, Wisconsin, United States of America; 5 Department of Biomedical Physiology and Kinesiology, Simon Fraser University, Burnaby, Canada; 6 Division of General Internal Medicine, University of California, San Francisco, California, United States of America; 7 Department of Epidemiology, Center for Aging and Population Health, Univeristy of Pittsburgh, Pittsburgh, Pennsylvania, United States of America; 8 Deparment of Nutritional Science, National Institute of Biomedical Innovation, Health and Nutrition, Tokyo, Japan; 9 Laboratory of Epidemiology and Population Sciences, IRP, National Institute on Aging, National Institutes of Health, Bethesda, Maryland, United States of America; University of Georgia, UNITED STATES

## Abstract

**Background:**

Total daily energy expenditure (TEE) is composed of resting metabolic rate (RMR), post-prandial thermogenesis and activity energy expenditure (AEE). Higher AEE is strongly associated with lower mortality and physical limitations among older adults, but factors that predict changes in AEE in septu and octogenarians are not clearly understood.

**Objective:**

To identify factors associated with late-life longitudinal change in AEE.

**Design:**

Energy expenditure was re-assessed in 83 participants (average age at baseline, 74.4±3.2 years)—an average of 7.5±0.54 years since the baseline measure. RMR was measured using indirect calorimetry and the thermic effect of meals was estimated at 10% of TEE. AEE was calculated as: TEE(0.9)-RMR. Participants were categorized into two groups according to the estimated day-to-day precision of the doubly-labeled water technique. Those who were within 10% or increased relative to their initial AEE measurement were categorized as having preserved AEE. Participants who declined greater than 10% of their initial measurement were categorized as having reduced AEE. A variety of socio-demographic, functional and mental factors, body composition, community and personal behaviors, blood measurements and health conditions were evaluated between groups at baseline and changes during follow-up.

**Results:**

Daily AEE declined 106.61±293.25 kcal, which equated to a 14.63±40.57 kcal/d decrease per year. Fifty-nine percent (n = 49) preserved their AEE and 41% (n = 34) declined. Those who demonstrated a decline in AEE were older, had lower walking speed at baseline and showed a higher lean mass loss during follow up. Otherwise, groups were similar for socio-demographic characteristics, body composition, mental and physical function, health conditions and community and personal behaviors at baseline and change in these factors during follow-up.

**Conclusions:**

This study demonstrates that AEE declines through the 8^th^ decade of life and is associated with age, lower walking speed at baseline and lean mass loss. Additionally, there are a significant number of individuals who appear to be resilient to these declines despite having health events that are expected to have a negative impact on their physical activity.

## Introduction

Total energy expenditure (TEE) consists of three components: resting metabolic rate (RMR), diet-induced thermogenesis or energy due to the thermic effect of food and activity energy expenditure (AEE). AEE is primarily due to muscular activity and is the most variable component of TEE [1, 2]. It is composed of both volitional exercise (e.g., walking or jogging) for exercise of leisure and non-exercise activity thermogenesis (NEAT)—standing, walking, occupational and household tasks. Previous studies have demonstrated that greater AEE is associated with reduced mortality [3], mobility limitation [4] and incidence of cognitive impairment [5] among older adults. However, while there is accumulating evidence of the health benefits of AEE, the factors that are associated with longitudinal changes in AEE have yet to be clearly identified.

Aging is associated with decreases in TEE that results from declines in both RMR and AEE [6, 7], although the latter seems to explain disproportionately more of this decrease [8, 9]. Aging is also associated with changes in body composition and comorbidities that lead to cognitive [10] and physical impairments [11, 12]. For example, demented older adults have extremely low AEE levels [6] and physical impairments are known to negatively impact AEE [13]. Additionally, we have noted that energy requirements in late life decline in men greater than that seen in women [14]. However, most studies have been cross-sectional which might lead to misinterpretation of the actual changes in AEE that occur within an individual. Moreover, the existing longitudinal studies have typically examined AEE changes in the younger-old population (<70 years). Some evidence suggests that the decline after 75 years old is steeper, yet this has not been fully confirmed [15, 16]. Declines in AEE are likely to result from a variety of medical and personal factors that relate to both personal and environmental changes that impact activity levels [17].

We sought to understand how a variety of factors (socio-demographic, body composition, diseases & hospitalization, participation in community activities, blood glucose, inflammatory biomarkers and vitamin D), are associated with longitudinal changes in AEE among septuagenarians who were assessed using doubly-labeled water at two time points separated by an average of 7.5±0.54 years. Such information would be helpful in identifying targets to preserve AEE that could conceivably lead to better health among older adults. Therefore the objective of this study was to comprehensively explore personal, anthropometric, mental function, medical events and physical function factors that might explain longitudinal changes in AEE among community-dwelling older men and women. We hypothesized that worsening health (e.g. disease, physical and cognitive impairments) and or personal factors are significant contributors to AEE decline.

## Materials and methods

### Study sample

The institutional review boards at the University of Pittsburgh and University of Tennessee, Memphis approved this study. Written informed consent, approved by the institutional review boards at the University of Pittsburgh and University of Tennessee, Memphis, was obtained from each participant.

**Fig 1:**
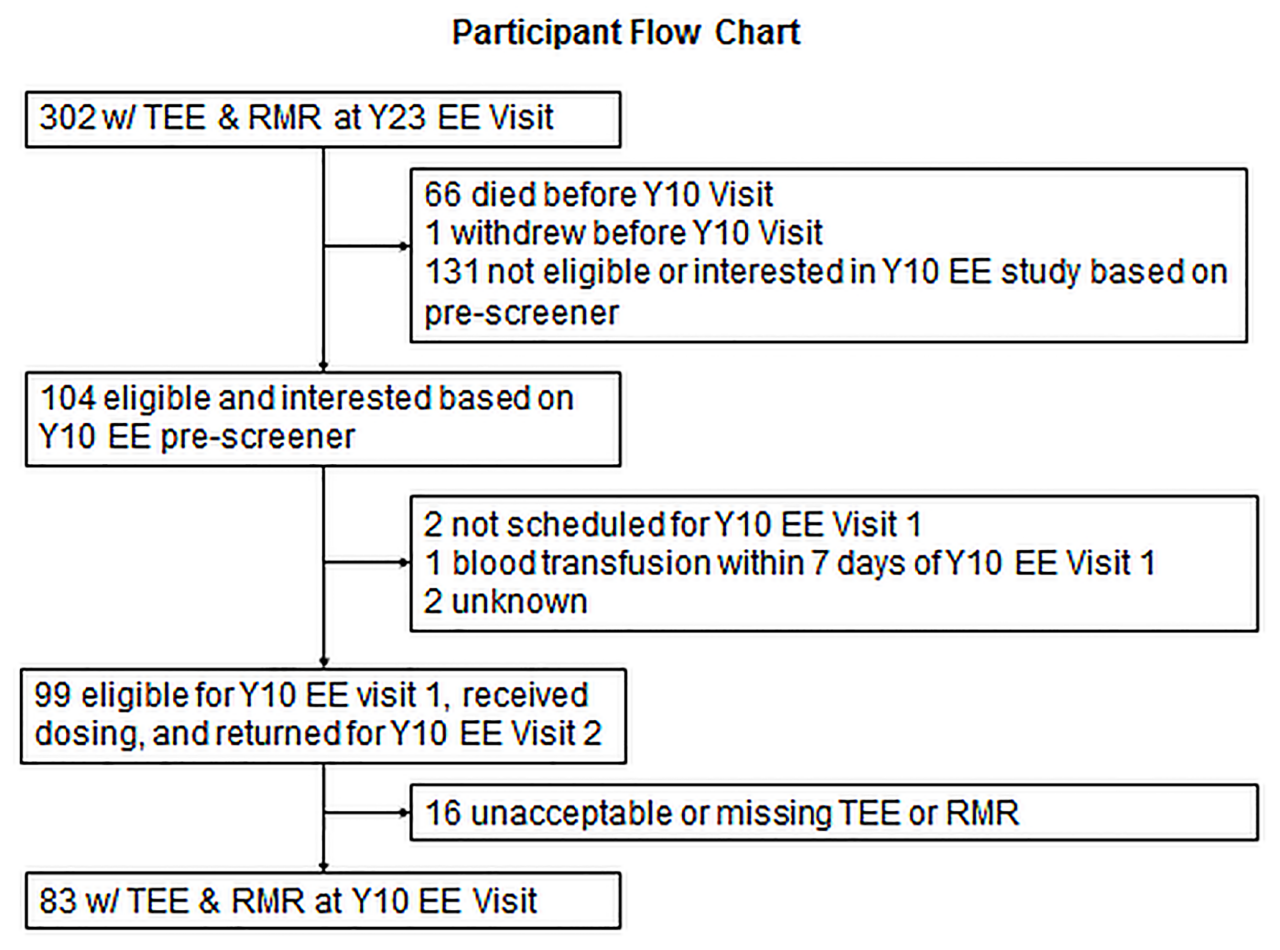
Consort diagram of participant flow for follow-up (FU) doubly-labeled water (DLW) visit.

In 1997–98 investigators from the University of Pittsburgh and University of Tennessee, Memphis, recruited 3075 participants aged 70–79 from a random sample of white Medicare beneficiaries and all age eligible self-identified black community residents to participate in the Health, Aging and Body Composition (Health ABC) study. Eligibility criteria included self-reporting no difficulty walking ¼ mile (0.4 km), climbing 10 stairs, or performing activities of daily living, no plans to leave the area for the next three years, and no evidence of life-threatening illnesses. The sample was approximately balanced for sex (51% women) and 42% of participants were black. Written informed consent, approved by the institutional review boards at the University of Pittsburgh and University of Tennessee, Memphis, was obtained from each participant. A participant flowchart of the energy expenditure (EE) sub-study, first carried out between 1998 and 2000, is illustrated in Fig 1.

A randomly selected list of 500 participants stratified by race and sex was generated from study eligible individuals, those who did not have a: recent blood transfusion, use of supplemental oxygen or insulin, and plan overnight travel immediately before or during the EE sub-study. A total of 323 participants were enrolled (n = 92 in 1998, n = 125 in 1999 and n = 85 in 2000) at baseline. Twenty-one participants were excluded at baseline because of failure to complete the protocol, lack of adequate urine volume specimens, or failure of isotope or RMR data to meet a priori quality control criteria. The baseline sample was of 302 participants (150 men and 152 women).

In 2006–2007, participants in the original sample were re-contacted and asked to participate in a follow-up EE sub-study. The breakdown of eligible and interested participants is illustrated in Fig 1. In brief, 66 died, 1 withdrew and 131 were not eligible at follow-up (FU) visit because of pre-specified criteria listed above. Out of the 104 that were eligible, 99 returned for doubly-labeled water (DLW) dosing. Eight-three participants had complete valid energy expenditure data and served as the sample for the current analysis.

### General overview of the doubly-labeled water protocol

Participants completed the DLW protocol with 2 visits to the clinic, each time arriving in a fasted state. During visit 1, participants received a dose of DLW for measurement of total energy expenditure (TEE) according to a protocol previously described [18, 19]. During this visit, body composition measures were ascertained using dual-energy X-ray absorptiometry (DXA). Participants returned to the clinic for a second visit fourteen days (14±1 days) later, where their body weight and RMR was measured. Two additional urine samples were collected for the endpoint DLW analysis. Participants were encouraged to maintain their normal activity levels between visit 1 and 2.

### Total energy expenditure (TEE)

Total energy expenditure (TEE) was measured using the 2-point DLW technique that has been previously described in detail [18]. Briefly, on the first visit, participants ingested 2 g/kg estimated total body water (TBW) dose of DLW, composed of 1.9 g/kg estimated TBW of 10% H_2_^18^O and 0.12 g/kg estimated TBW of 99.9% ^2^H_2_O. After dosing, three urine samples were obtained at approximately 2, 3, and 4 hours. Two consecutive urine voids were taken during a second visit to the laboratory, approximately fourteen days after the first visit. Plasma from a 5 mL blood sample was obtained from everyone but only used for those who had evidence of delayed isotopic equilibration likely caused from urine retention in the bladder (n = 28)^18^. Urine and plasma samples were stored at –20°C until analysis by isotope ratio mass spectrometry.

Dilution spaces for ^2^H and ^18^O were calculated according to Coward [20]. Total body water was calculated as the average of the dilutions spaces of ^2^H and ^18^O after correction for isotopic exchange (1.041 for ^2^H and 1.007 for ^18^O). Carbon dioxide production was calculated using the two-point DLW method outlined by Schoeller and colleagues [21, 22] and TEE was derived using Weir’s equation [23]. A food quotient of 0.86 was used from the third National Health and Nutrition Examination Survey [24] and from Black and coworkers [25]. All values of energy expenditure were converted to kilocalories per day (kcal/d) and the thermic effect of meals was assumed to be 10% of TEE [26]. For measurement of total body water, the intra-subject repeatability calculated as the average percent difference between the two analyses, was -0.1 ± 1.2%. The intra-tester repeatability of TEE based on blinded, repeat, urine isotopic analysis was excellent (mean difference = 1.2±5.4%, n = 16).

### Resting metabolic rate (RMR)

Resting metabolic rate (RMR) was measured via indirect calorimetry on a Deltatrac II respiratory gas analyzer (Datex Ohmeda Inc., Helsinki); detailed procedures have been described elsewhere [19]. While in a fasting state and after 30 minutes of rest, a respiratory gas exchange hood was placed over the participant’s head and RMR was measured minute-by-minute for 40 minutes. To avoid gas exchange created by the initial placement of the hood, only the final 30 minutes were used in subsequent calculations. Movement or sleeping during the test was noted and those time periods were excluded from the RMR calculation. Methanol burn tests were performed in duplicate once or twice per month. Carbon dioxide recovery averaged 100.1±1.4% at the Pittsburgh site and 100.5±1.5% at the Memphis site. The gas exchange ratios for methanol differed by 2.5% between sites (Pittsburgh: 0.68±0.01, Memphis: 0.66±0.01, p < 0.001) and this difference did not demonstrate a trend over time. Therefore, a correction factor was employed to equate the two study sites by dividing the respiratory ratios for participants enrolled at Pittsburgh by 1.025.

### Activity energy expenditure (AEE)

To calculate AEE, the thermic effect of food was assumed to be 10% of TEE in the equation: AEE = (TEE*0.9)-RMR [21, 22]. AEE is defined as the calories an individual expends in any and all activities per day.

### Predictors

#### Socio-demographic characteristics

Age, gender, race (white/black), marital status (married/was married/never married), study site (Memphis/Pittsburgh) and education (high school vs non high school) were assessed during the baseline clinic visit.

#### Prevalent or incident disease and hospitalization

Self-reported medical conditions with confirmation by treatment and or medication [cardiovascular disease (coronary heart disease, myocardial infarction, hypertension and stroke), lung disease, diabetes, hip or knee osteoarthritis, osteoporosis, cancer, fractures, falls and depression] were measured at baseline and the follow-up visit. Hypertension was defined by use of an antihypertensive medication or measured systolic blood pressure exceeding 140 mmHg or diastolic blood pressure exceeding 90 mmHg. Diabetes was defined by use of diabetes drug or fasting plasma glucose >126 mg/dL or 2-hour post-challenge glucose >200 mg/dL. Incident disease was determined at 6-month intervals with a phone or clinic visit. A reported hospitalization or health event was followed with collection of medical records and standardized adjudication procedures.

#### Mental and physical health

Physical function was measured using an established performance battery described previously [27]. The battery was a modified version of three lower-extremity performance tests used in the Established Populations for the Epidemiologic Studies of the Elderly (EPESE) [28] consisting of 5 repeated chair stands, standing balance (semi- and full-tandem stands), and a 6-m walk to determine usual gait speed. The holding time of the semi- and full-tandem stands was increased to 30 seconds and a 30-second single leg stand was added to the standing balance test. A narrow walk test of balance was also added. Participants received a score that ranged from 0–12 with zero being the poorest function. Isometric grip strength was measured with a hand-held dynamometer (JAMAR Technologies, JLW Instruments, Chicago, IL) and the maximum grip strength in kilograms after two attempts with either hand was used. Gait speed was assessed with participants instructed to walk at their usual pace over a 20-m course. Center for Epidemiologic Studies Depression (CES-D) scale was used to measure depressive symptoms. The Teng Modified Mini-Mental State Examination (3MS) was administered to participants at baseline and follow-up clinic visits [29]. Possible scores range from 0 to 100 with higher scores indicating better cognitive function. Self-rated health was categorized into fair and poor health using a standardized questionnaire. Mobility limitation was assessed by using semi-annual questionnaires about walking ability: persistent mobility limitation was defined as 2 consecutive reports of any difficulty walking one-quarter of a mile; persistent lower extremity limitation was defined as 2 consecutive reports of either having any difficulty walking one-quarter of a mile or having any difficulty walking up 10 steps without resting. To qualify, the consecutive reports must involve the same function (i.e. two walking or two stairs not one walking followed by one stairs).

#### Anthropometry & body composition

Dual-energy x-ray absorptiometry (QDR-4500, version 8.21, Hologic Inc, Bedford, Mass) was used to determine fat mass and lean mass as described previously [30]. Lean mass was calculated by removing mass due to bone mineral content. Body weight was measured in a hospital gown with no shoes using a calibrated balance beam scale and height was measured with a calibrated stadiometer.

#### Community and personal behaviors

Physical activity over the past 7 days was assessed by an interviewer-administered questionnaire at the time of the doubly labeled water dosing. The questionnaire was modified from the College Alumnus Physical Activity Questionnaire to include tasks more applicable to older adults [31]. Questions about walking for exercise, other walking, climbing stairs, working for pay, and volunteering were assessed. The smoking status (not smoking or current), alcohol use, the loss of the spouse or a relative, time watching television, appetite, and sleeping difficulty were also evaluated using standard questionnaires.

#### Blood measures

Fasting glucose, Hb1Ac (glycated hemoglobin), CRP (C-reactive protein) and IL-6 (interleukin six) were measured at baseline and during the follow up visits. Vitamin D was measured only at baseline.

Fasting glucose was measured on a Johnson and Johnson Vitros 950 analyzer, HbA1c was measured using Tosoh 2.2 Plus (Tosoh Bioscience, Tokyo, Japan) as described previously [32]. CRP levels were measured in duplicate by enzyme-linked immunosorbent assay (ELISA) based on purified protein and polyclonal anti-CRP antibodies (Calbiochem, San Diego, CA). Standardization was done using the WHO International Reference Standard with a sensitivity of 0.08 mg/L. The lower limit of detection for CRP was 0.007 mg/L. IL-6 was determined by ELISA measured in duplicate using a high-sensitivity Quantikine colorimetric immunoassay kit from R&D Systems (Minneapolis, MN) with a detectable limit of 0.10 pg/mL. Vitamin D or serum 25(OH)-D was analyzed by a two-step radioimmunoassay kit (DIASORIN, 25-hydroxyvitamin D 125I RIA kit, no. 68100, Stillwater, MN) as explained previously [33].

### Data analysis

**Fig 2:**
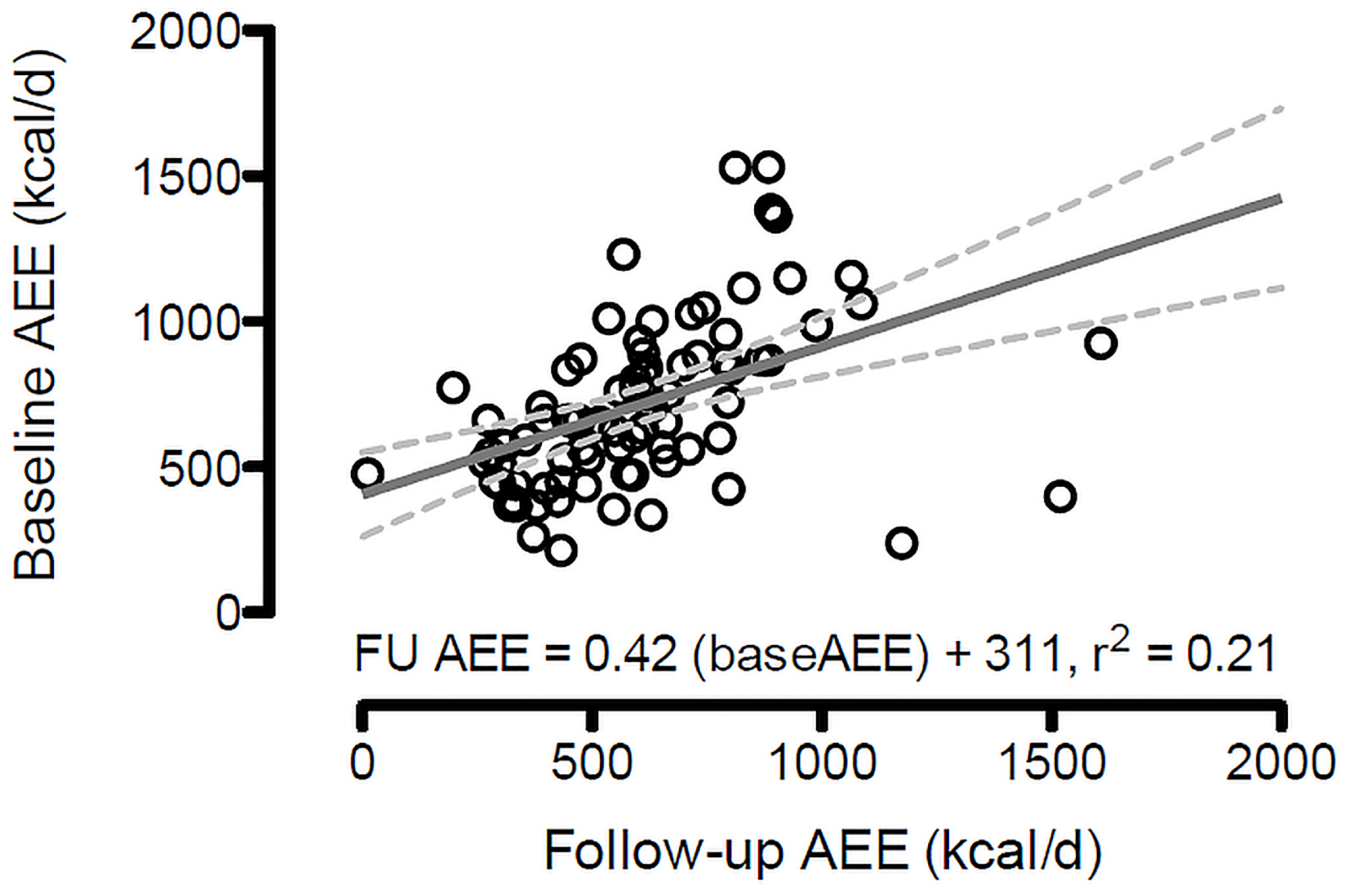
Scatterplot and line of best fit for baseline and follow-up activity energy expenditure.

As illustrated in Fig 2, baseline AEE is highly correlated with follow-up AEE (r = 0.46, p<0.01). When two variables are correlated, as observed with baseline and follow-up AEE, the calculated difference score (i.e. change score) is often spuriously negatively correlated with baseline values. [34, 35]

In this study, AEE changes were highly correlated with baseline AEE values (r = -0.58, p<0.01). To adjust for baseline variance, residualized values were created using a linear regression of follow-up on baseline values. Residualized values are referred to as “baseline-free” measures of change that help combat “regression to the mean” in change analyes [36, 37, 38]. There were two steps in creating change groups. First, the residulaized values were divided by an individuals’ predicted value and multiplied by 100 to yield a percent of predicted difference. We then opted to group individuals according to a threshold that exceeds methodological error of the DLW technique which is a within-subject coefficient of variation of 7.8% for TEE [39]. We conservatively chose a 10% difference threshold to categorize participants. Participants with a greater than 10% reduction from their predicted baseline value were categorized as having a meaningful decrease in AEE. Those who had less than a 10% reduction were considered to have maintained their AEE. Comparisons were then made between participants who declined versus maintained their AEE as compared to what would be predicted from their baseline AEE. Comparisons were also made at baseline between participants who were lost to follow-up and those who completed the DLW follow-up visit. Chi-square statistics were used to test group differences for categorical variables and analysis of variance for continuous variables.

Changes in body composition, 3MS score, CES-D score, physical performance score, walking speed, grip strength, mobility limitation, blood measures and time spent in self-reported activity for each participant over time was calculated and compared between participants who declined versus maintained their AEE. Comparisons between the two AEE groups (maintained vs declined) were also made for new acquired conditions, new hospitalizations and number of new falls using Wilcoxon rank-sum test for non-parametric data.

Stata statistical software version 11.0 (StatCorp, College Station, Tex) was used for all analyses and results were considered statistically significant at p < 0.05.

## Results

**Fig 3:**
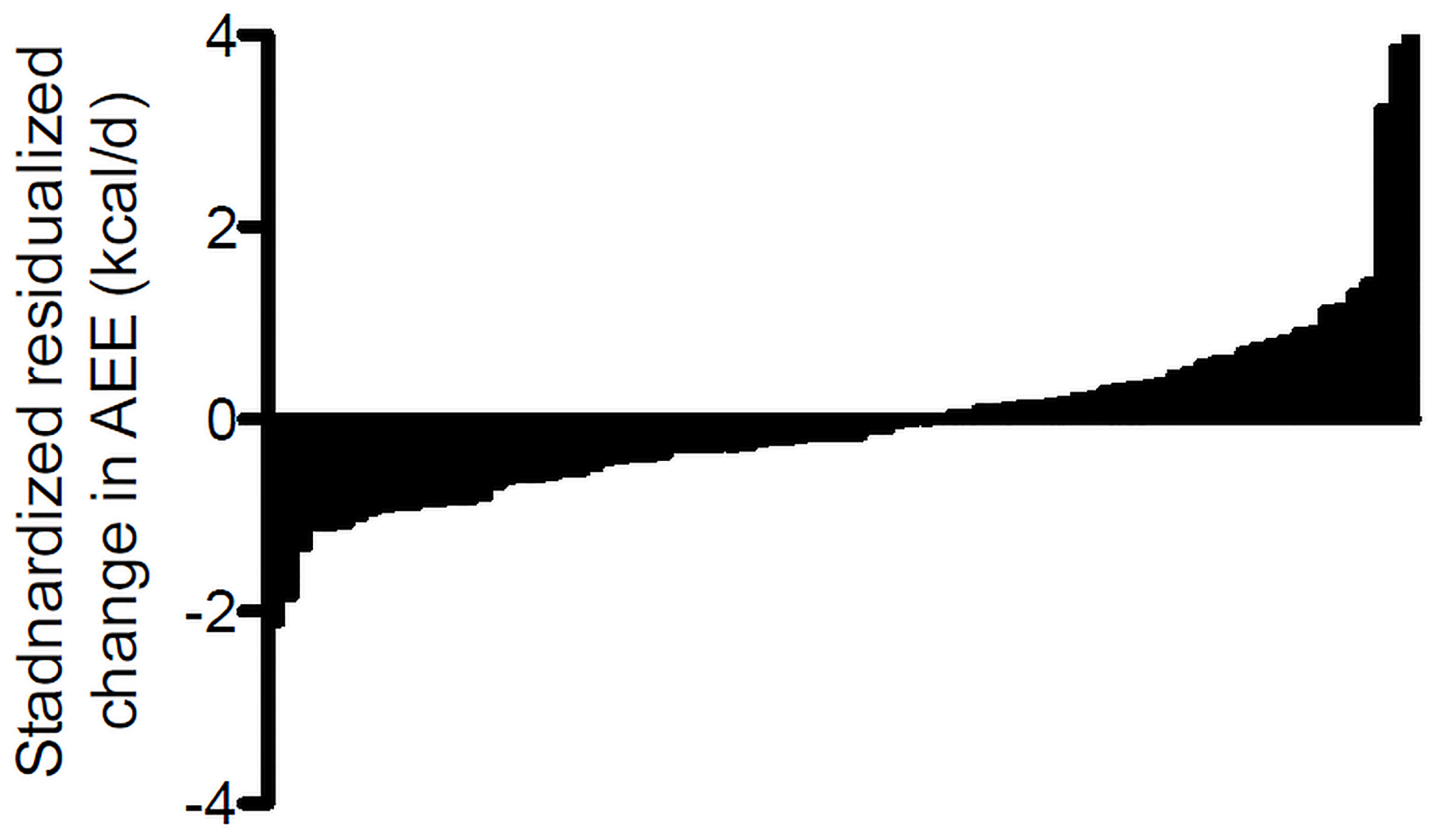
Distribution of follow-up AEE values adjusted for baseline. Values are AEE residuals after regression baseline AEE on follow-up AEE. Values are standardized to a mean of zero.

At baseline, participants who were lost to follow-up (N = 219) were similar in terms of age, gender, race, body composition, depression score, and 3MS score when compared to participants who completed the follow-up assessment (S1 Table). However, those who were lost to follow-up had a lower physical performance score (p = 0.002), were more likely to have cancer (p = 0.022), were less likely to volunteer their time (p = 0.023), and reported less time walking for exercise (p = 0.03). Participants completed the follow-up evaluation 7.5±0.54 years from their baseline visit. On average, there was 106.61±293.25 kcal/d decrease in AEE, which equated to a 14.63±40.57 kcal/d/year decline or 2.05% decline per year. Of the 83 participants, 49 (59%) maintained their AEE within 10% of their baseline during the follow-up and 34 (41%) had greater than a 10% reduction in AEE (Fig 3 illustrates the distribution of standardized AEE residuals).

Those categorized as having a decline in AEE demonstrated a 239.57±192.30 kcal/d reduction (37.3% p < 0.001). Participants categorized as maintaining their AEE showed a -14.35±316.65 kcal/d (1.9% p < 0.001) reduction.

Very few baseline differences were noted between participants experiencing a decline versus those who maintained their AEE (See Table 1 for comparisons).

**Table 1:** Baseline characteristics for two groups of participant who maintained or declined over an average of 7.5 years of follow-up.

Characteristics	Declined AEE (N = 34)	Maintained AEE (N = 49)	P-value
**Socio-demographic characteristics**			
Site (Memphis), N (%)	15 (44.1)	23 (46.9)	0.800
Age (yr)	75.5 (3.0)	73.7 (3.1)	**0.008**
Female, N (%)	16 (47.1)	22 (44.9)	0.846
Black, N (%)	16 (47.1)	20 (40.8)	0.573
Living alone, N (%)*	9 (26.5)	9 (18.8)	0.405
High school education, N(%)	26 (76.5)	35 (71.4)	0.609
**Prevalent disease**			
Cardiovascular disease N (%)	9 (26.5)	9 (18.4)	0.378
Diabetes N (%)	6 (17.7)	4 (8.2)	0.192
Cancer N (%)	8 (23.5)	9 (18.4)	0.567
Osteoarthritis N (%)	3 (8.8)	4 (8.2)	0.915
Lung disease N (%)	3 (8.8)	4 (8.2)	0.915
Osteoporosis N (%)	5 (14.7)	3 (6.1)	0.193
Diagnosed depression N (%)	7 (20.6)	6 (12.2)	0.304
Hospitalizations prior to baseline	1 (0)	1.3 (0.7)	0.263
**Body mass & composition**			
Body weight, kg	75.6 (12.3)	77.9 (13.1)	0.423
Body mass index, kg/m^2^	27.8 (4.8)	27.8 (4.4)	0.985
Percentage of body fat	33.6 (8.4)	32.9 (7.9)	0.710
Lean mass, kg	47.7 (9.0)	49.7 (9.7)	0.344
**Blood measures**			
Fasting glucose (mg/dl)	97.1 (20.2)	94.1 (20.3)	0.516
A1c (%)	6.3 (1.4)	6.0 (0.5)	0.162
CRP (ug/ml)	6.53 (10.02)	6.58 (14.44)	0.986
IL-6 (pg/ml)	3.08 (3.10)	3.42 (5.62)	0.754
Vitamin D (ng/ml)	26.2 (9.5)	26.6 (9.6)	0.821
**Mental and physical health**			
Cognition score on 3MS	89.3 (7.9)	91.4 (6.9)	0.199
Depression on CES-D	4.2 (3.3)	3.8 (2.8)	0.568
Physical performance score	7.0 (1.4)	7.5 (1.3)	0.129
Grip strength (kg)	33.9 (11.0)	34.9 (10.5)	0.666
Long distance walking speed (m/s)**	1.25 (0.19)	1.35 (0.19)	**0.041**
20-m usual walking speed (m/s)	1.13 (0.21)	1.23 (0.22)	**0.039**
20-m rapid walking speed (m/s)	1.54 (0.27)	1.67 (0.26)	**0.036**
Self-rated fair or poor health N (%)	18 (52.9)	22 (44.9)	0.471
Persistent mobility (walking) limitation N (%)	6 (17.7)	6 (12.2)	0.491
Persistent lower extremity limitation N (%)	7 (20.6)	8 (16.3)	0.620
**Community and personal behaviors**			
Working, N (%)	6 (17.7)	16 (32.7)	0.128
Volunteering, N (%)	15 (44.1)	30 (61.2)	0.124
Self-reported walking minutes/week	147.7 (203.1)	111.1 (190.8)	0.406
Time watching TV: >14 hours/week, N (%)	19 (55.9)	17 (34.7)	**0.055**
Time reading, hours/wk	10.8 (8.9)	12.9 (9.6)	0.316
Appetite (very good), N (%)	16 (47.1)	24 (49.0)	0.863
**Energy expenditure at baseline (kcal/d)**			
Total	2085 (442)	2295 (521)	0.059
Resting	1234 (209)	1300 (226)	0.182
Activity	643 (267)	766 (307)	0.063
**Change in activity energy expenditure (AEE)**			
Raw change in AEE (FU—baseline AEE) (kcal/d)	-239 (192)	-14.3 (316)	
Predicted FU AEE based on baseline AEE (kcal/d)	578 (110)	629 (127)	
Residualized AEE (kcal/d)	-175 (101)	121 (228)	
Residualized AEE as a percent of predicted (%)	-30.8 (18.3)	21.8 (44.6)	

Abbreviations: FU, follow-up; A1c, glycated hemoglobin; CRP, C-reactive protein; IL-6, interleukin-six; 3 MS, Modified Mini Mental State Examination; CES-D, Center for Epidemiologic Studies Depression.

*Nine participants did not answer the question about living status: total evaluated = 75 participants.

**Twenty-two participants did not complete the long distance walking test at baseline: total evaluated = 61 participants.

Participants with a reduction in AEE were older and had a slightly lower walking speed on 400 meter walk test and on 20 meter walk test at both usual and rapid pace.

Table 2 shows the comparison between the two AEE groups with regard to new acquired diseases and surgeries, new hospitalizations, new falls, changes in mental and physical health, body composition, mobility limitation and community and personal behaviors.

**Table 2:** Change in predictors between participants who maintained or declined over an average of 7.5 years of follow-up.

Characteristics	Decline AEE (N = 34)	Maintain AEE (N = 49)	P-value
**New health conditions**			
Acquired* diagnosed diseases (and fracture)	4.4 (2.3)	5.4 (2.5)	0.070
Number of new surgeries	1.6 (1.8)	1.3 (1.9)	0.575
Number of new hospitalizations	2.6 (2.8)	2.0 (2.3)	0.238
Length of stay (LOS)/number of hospitalizations	4.0 (3.5)	3.0 (3.1)	0.159
Number of new falls	2.3 (2.6)	3.2 (5.3)	0.349
Restricted activity or bedrest (n. of events)	3 (2.6)	2.8 (2.7)	0.758
Number days bedrest	3.5 (4.7)	4.5 (8.1)	0.535
**Blood measures**			
Fasting glucose (mg/dl)	0.64 (17.83)	3.61 (16.67)	0.453
A1c (%)	0.40 (0.45)	0.38 (0.78)	0.887
CRP (ug/ml)	1.89 (5.97)	-0.14 (10.25)	0.330
IL-6 (pg/ml)	0.49 (1.78)	0.67 (4.55)	0.837
**Body mass & composition**			
Body mass, kg	-3.8 (6.1)	-1.9 (5.1)	0.122
Percent body fat, kg	0.9 (2.9)	0.7 (3.1)	0.838
Fat free mass, kg	-0.4 (3.5)	1.5 (2.7)	**0.009**
Lean mass, kg	-3.0 (2.8)	-1.7 (2.5)	**0.023**
Appendicular lean mass, kg	0.06 (1.43)	0.53 (1.25)	0.136
**Mental and physical health**			
Change in cognition score on 3MS	-2.4 (6.7)	-0.9 (5.1)	0.240
Change in CES-D	1.6 (4.2)	0.7 (2.5)	0.231
Change in physical performance score	-1.9 (1.7)	-1.6 (1.6)	0.389
Change in grip strength	-4.44 (5.54)	-4.40 (3.73)	0.966
Change in long distance walking speed**	-0.18 (0.16)	-0.17 (0.14)	0.911
Self-rated fair or poor health N (%)	9 (26.5)	7 (14.3)	0.166
**Community and personal behaviors**			
Death of a family member or spouse, N (%)	24 (70.6%)	39 (79.6%)	0.346
Stop in working/ volunteering status, N (%)	7 (33.3)	20 (57.1)	0.084
Change in self-reported walking minutes	-62.4 (185.5)	-26.3 (161.7)	0.349
**Energy expenditure change (kcal/d)**			
Total	-318 (264)	-81 (370)	**0.002**
Resting	-46 (133)	-59 (107)	0.639
Activity	-240 (192)	-14.3 (317)	**<0.001**

Abbreviations: A1c, glycated hemoglobin; CRP, C-reactive protein; IL-6, interleukin-six; 3 MS, Modified Mini Mental State Examination; CES-D, Center for Epidemiologic Studies Depression.

*Numbers of incident (new) conditions in the past 12 months from the second DLW visit.

**Forty-one participants did not have the measure of change of long distance walking test: total evaluated = 42 participants.

After 7.5 years of follow up, participants with a reduction in AEE showed a significant decrease in lean mass. The two groups showed similar changes in number of hospitalizations, falls, incident disease or surgery, community and personal behaviors, 3MS score, and CES-D score compare to those who maintained AEE.

## Discussion

This study explored a variety of inter and intra individual factors that were associated with meaningful longitudinal change in daily activity energy expenditure (AEE), using the doubly labeled water (DLW) method. On a whole, AEE decreased by 106.61±293.25 kcal/day over an average of 7.5±0.54 years in older adults. However, there was significant variability in AEE change with 59% of the cohort appearing to have preserved AEE. The current analysis examined other environmental and personal factors that would contribute to changes in AEE in late-life. Significant AEE change predictors included age, walking speed at baseline and change in lean mass during follow up. Interestingly, acquired conditions and catastrophic health events did not impact AEE change over the follow-up period. Overall, the data suggest we have a limited knowledge regarding the factors associated with AEE change in late—life.

### Aging and activity energy expenditure

Several cross-sectional studies have added to our current understanding of age-related changes in AEE. A large cross-section study by Black and colleagues [6] found that AEE was negatively associated with age in a sample of participants aged 2 to 95 years. Specifically, from adolescence to ~ 75 years there is an approximately 0.57% kcal/day/year decline in AEE and this decline appears greater in men (60%) than women (47%). The current study also noted a trend toward women being more likely than men to maintain their AEE. Another cross-sectional study by Johannsen and colleagues showed similar rate of change when comparing young (20–34 years) to older adults (60–74 years) [40]. Interestingly, adults in their nineties experienced a 0.69% and 0.96% kcal/day/year AEE decline in men and women, respectively, suggesting an accelerated decline in very late life. The sex difference and accelerated decline of AEE change are consistent with the results of sex difference in age-related decrease of biological and functional age and accelerated decline of biological and functional age [41, 42].

The current study is among the few examining longitudinal changes in AEE in older adults. To our knowledge, only one study assessed longitudinal changes in free-living AEE among old individuals [15]. Over 5 years, Rothenberg and coworkers found a non-significant 122 kcal/d (or 2.5% kcal/day/year) decrease in AEE among eleven people in their seventh decade [15]. Comparing to the current study, the mean AEE at baseline was substantially higher (967.35 kcal/d compared to 715 kcal/d in the current study), reflecting a more physically active and potentially healthier sample. In general, our results show a 2.0% decline in AEE per year, suggesting a slightly smaller decline as compared to the previous longitudinal study, but a larger decrease compared to the cross-sectional studies. Coupling the current findings with those previously mentioned suggests that AEE declines more rapidly in late life.

### Predictors of late-life change in daily activity energy expenditure

Fifty-nine percent of the particiapnts maintained their AEE within measurement error and 41% demonstrated a decline after an average of 7.5 years of follow up. These two groups who represent a relatively large sample size for a DLW longitudinal study provided an opportunity to explain AEE changes. The literature suggests that there are multiple factors that contribute to AEE changes such as those originating from biological, psychological, socio-demographic, medical and environmental causes [17]. For example, poor health status, measured by the presence of chronic conditions [43], self-reported health status [44, 45], depression [46, 47] and disability [48] is consistently associated with lower levels of physical activity. In this longitudinal study, some of this previous work was confirmed (walking speed), but others such as having a high rate of comorbidities or newly acquired comorbidities were not. Subsequent paragraphs discuss and interpret the most significant explanatory domains.

#### Acquired medical conditions and hospitalizations

AEE levels are well known to be lower in individuals with active symptoms for heart failure [49, 50], Parkinson’s disease [51] and lung cancer [52]. Less well studied are catastrophic events like hospitalizations [53] and falls [54] that are theoretically associated with reduction in AEE. Therefore, we expected that newly acquired medical conditions and catastrophic events would negatively impact AEE change. However, despite a relatively high rate of newly acquired conditions and events, there were no differences between the individuals who had maintained or showed declines in their AEE. Although it should be noted there was a trend for hospitalizations, days with restricted activity and days on bed rest to be more prevalent in individuals with decline in AEE that is consistent with our hypothesis.

#### Body mass & composition

Participants with a reduction in AEE showed a significant higher loss of fat free mass and lean mass after 7.5 years of follow up compared to those who maintained their AEE. Although physical activity is likely to have a role in preventing lean mass loss, existing cross-sectional and longitudinal studies have produced controversial results. In particular, two previous reports showed that current body composition and change in body composition, especially fat free mass, had little association with AEE change [55, 56]. On the other hand, a recent cross-sectional research study demonstrated that higher objectively assessed AEE is associated with higher lean mass in early old age [57] and another longitudinal study showed that greater physical activity retained a greater lean mass over 5 years of observation [58]. Our findings confirm these results and suggest that age-related decline in AEE is associated with reduction in both free fat and lean mass.

#### Physical and functional status

Among physical and functional factors, only baseline walking speed on the 400 meter walk test and the 20 meter walk test at usual and rapid pace were significantly associated with declines in AEE. These findings are supported by a wealth of literature suggesting that lower walking speed acts as a major impediment to physical activity [59, 60, 61, 62, 63]. Although there is less work on the impact that walking speed has on AEE, one study found that higher levels of AEE were associated with both faster walking speed and lower risk of mobility limitation [4].

#### Community and personal behaviors

A variety of community and personal behaviors were examined, but none of these behaviors appeared to be related to AEE changes. Based on the previous literature, self-reported walking behavior was certainly expected to be a major predictor [64], but there was a high level of variability in walking behavior made detectable differences difficult to observe. Interenstingly, the proportion of indviduals watching excessive TV—a sedentary behavior [65],—was slightly higher among group demonstrating a decline in AEE. Regarding other factors, behaviors, difficulty sleeping [66], appetite [67] and volunteering [68] have theoretical connections to AEE, but these were not verified in the current study.

### Limitations

There are several limitations that should be recognized. First, while this study represents one of the largest longitudinal studies on changes in AEE components among older adults, only about a third of the initial sample completed the follow-up measurements. This presents a potential healthy participant and or survivorship bias. Those who completed the evaluation had better cognition, higher physical performance scores, were more likely to report good health and were more likely to volunteer their time. Consequently, the results are not completely representative of the older adult population. We chose to express AEE in its raw form while others have used the ratio of AEE and body weight to account for large body sizes. We examined this approach and found a strong correlation (r = 0.95) between residualized AEE and residuliazed AEE to body weight ratio, which ultimately demonstrated similar findings as the current results. Secondly, while our approach investigated numerous domains that were theoretically related to AEE change; it was not fully comprehensive for all the factors that are theoretically linked to changes in AEE. In addition, we did not measure physical activity movement (e.g. accelerometer) and/or posture allocation. The cost of locomotion or sedentary duration (sit to stand ratio) might be changed even AEE did not change [69, 70].

## Conclusions

An interesting observation in this study was the large variability in AEE changes—measured by doubly labeled water—over an average of 7.5 years in septu and octogenarians. This variability was partially explained by having older age, lower walking speed at baseline and higher free fat and lean mass loss during follow-up. Socio-demographics characteristics, other body composition characteristics, community and personal behaviors, acquired medical conditions and catastrophic health events were surprisingly not associated with AEE changes. The lack of robust predictors suggests that individuals who maintain their AEE are resilient to adversities that occur with aging. For example, there were a significant number of individuals who were resilient to age-related declines in AEE, despite having catastrophic health events and other personal and environmental behaviors that were expected to negatively impact activity levels. These older adults may have maintained a positive adaptation through physiological, social, emotional or spiritual coping strategies in the face of adversity that would positively influence their daily activity levels [71]. In conclusion, the results suggest that efforts to preserve walking speed and lean mass are expected to impact late-life longitudinal changes in AEE.

## Supporting information

S1 Table Baseline characteristics of participants who completed a doubly-labeled water follow-up evaluation and baseline characteristics over an average of 7.5 years. Abbreviations: SD, standard derivation; 3 MS, Modified Mini Mental State Examination; CES-D, Center for Epidemiologic Studies Depression; BMI, Body Mass index. Note: Individuals are categorized as completed if they underwent a second measure of doubly-labeled water and quality control was reached. Individuals who were lost to follow-up are those who did not undergo a second measure of doubly-labeled water or quality control was not reached. *Nine participants did not answer the question about living status: total evaluated = 293 participants for lost to follow-up versus completed and 75 participants for maintain versus decline.
(DOCX)

S1 Dataset Database of the study. (CSV)
