# Supplementary material for: Surveying predictors of late-life longitudinal change in daily activity energy expenditure
Source: PLoS One. 2017 Oct 17;12(10):e0186289. doi: 10.1371/journal.pone.0186289 (PMC5645098; doi:10.1371/journal.pone.0186289)
Supplement: S1 Table — Abbreviations: SD, standard derivation; 3 MS, Modified Mini Mental State Examination; CES-D, Center for Epidemiologic Studies Depression; BMI, Body Mass index. Note: Individuals are categorized as completed if they underwent a second measure of doubly-labeled water and quality control was reached. Individuals who were lost to follow-up are those who did not undergo a second measure of doubly-labeled water or quality control was not reached. *Nine participants did not answer the question about living status: total evaluated = 293 participants for lost to follow-up versus completed and 75 participants for maintain versus decline. (DOCX) [file pone.0186289.s001.docx]

**S1_Table.**

| **Characteristic** | **Completed**  **evaluation**  **(N = 83)** | **Death**  **(=66)** | **Non death**  **(=153)** | **p-value** |
| --- | --- | --- | --- | --- |
| **Socio-demographic characteristics** |  |  |  |  |
| Site (Memphis), N (%) | 38 (45.8) | 43 (65.2) | 82 (53.6) | 0.062 |
| Age (yr) | 74.4 (3.2) | 75.3 (3.0) | 74.8 (2.8) | 0.198 |
| Female, N (%) | 38 (45.8) | 29 (43.9) | 85 (55.6) | 0.179 |
| Black, N (%) | 36 (43.4) | 36 (54.6) | 74 (48.4) | 0.399 |
| Living alone, N (%) * | 18 (21.9) | 19 (28.8) | 38 (26.2) | 0.621 |
| High school education, N (%) | 61 (73.5) | 43 (65.2) | 112 (73.2) | 0.430 |
| **Prevalent disease, N (%)** |  |  |  |  |
| Cardiovascular disease | 18 (21.7) | 49 (74.2) | 88 (57.5) | **0.045** |
| Diabetes | 10 (12.1) | 9 (13.6) | 20 (13.1) | 0.956 |
| Cancer | 17 (20.5) | 6 (9.1) | 17 (11.1) | 0.068 |
| Osteoarthritis | 7 (8.4) | 4 (6.1) | 18 (11.7) | 0.385 |
| Lung disease | 7 (8.4) | 9 (13.6) | 17 (11.1) | 0.597 |
| Osteoporosis | 8 (9.6) | 8 (12.1) | 17 (11.1) | 0.885 |
| Diagnosed depression | 13 (15.7) | 5 (7.6) | 22 (14.4) | 0.295 |
| **Body mass & composition** |  |  |  |  |
| Baseline body mass, kg | 76.9 (12.8) | 77.0 (16.0) | 75.8 (17.0) | 0.835 |
| Body mass index, kg/m^2^ | 27.8 (4.5) | 27.1 (5.1) | 27.2 (5.1) | 0.622 |
| Percent body fat | 33.2 (8.0) | 32.3 (7.8) | 34.5 (8.2) | 0.146 |
| Fat free mass, kg | 48.8 (49.3) | 49.3 (10.5) | 47.0 (10.5) | 0.209 |
| Fat mass, kg | 25.6 (7.8) | 24.9 (8.8) | 26.4 (9.9) | 0.517 |
| **Mental and physical health** |  |  |  |  |
| Cognition score on 3MS | 90.5 (7.4) | 85.8 (11.2) | 89.4 (8.3) | **0.004** |
| Depression on CES-D | 3.9 (3.0) | 4.5 (4.1) | 4.4 (3.9) | 0.566 |
| Physical performance score | 7.3 (1.3) | 6.3 (1.5) | 6.9 (1.4) | **<0.001** |
| Grip strength | 34.5 (10.6) | 33.6 (9.6) | 32.1 (9.4) | 0.185 |
| 20 meters walking speed (usual) | 1.19 (0.22) | 1.07 (0.16) | 1.14 (0.21) | **0.002** |
| Self-rated fair or poor health (versus excellent, good or very good), N (%) | 11 (13.25) | 23 (34.9) | 29 (18.9) | **0.004** |
| Persistent mobility limitation (difficulty to walk one quarter of mile) | 12 (14.5) | 17 (25.8) | 25 (16.3) | 0.157 |
| **Community and personal behaviors** |  |  |  |  |
| Working, N (%) | 22 (26.5) | 14 (21.2) | 31 (20.3) | 0.532 |
| Volunteering, N (%) | 45 (54.2) | 19 (28.8) | 68 (44.4) | **0.008** |
| Self-reported physical activity (walking, minutes) | 126.1 (195.6) | 73.3 (135.0) | 86.5 (145.4) | 0.089 |
| Time watching TV: >14 hours/week , N (%) | 36 (43.4) | 35 (53.1) | 86 (56.2) | 0.166 |
| Time reading , hours/week | 12 (9.3) | 9.4 (7.1) | 11.9 (9.0) | 0.114 |
| Appetite (very good), N (%) | 43 (51.8) | 24 (63.4) | 72 (47.1) | 0.160 |
| Report of a sleeping difficulty, N (%) | 74 (89.2) | 56 (84.9) | 142 (92.8) | 0.185 |
| Current smoker, N (%) | 7 (8.4) | 11 (16.7) | 16 (10.5) | 0.260 |
| **Energy expenditure at baseline (kcal/d)** |  |  |  |  |
| Total | 2209 (498) | 2141 (491) | 2148 (424) | 0.572 |
| Resting | 1272 (220) | 1305 (219) | 1263 (227) | 0.433 |
| Activity | 715 (295) | 621 (327) | 670 (261) | 0.139 |
